# Colorectal cancer incidence after the first surveillance colonoscopy and the need for ongoing surveillance: a retrospective, cohort analysis

**DOI:** 10.1136/gutjnl-2024-334242

**Published:** 2025-04-05

**Authors:** Emma C Robbins, Kate Wooldrage, Matthew D Rutter, Andrew M Veitch, Amanda J Cross

**Affiliations:** 1 Cancer Screening and Prevention Research Group (CSPRG), Department of Surgery and Cancer, Imperial College London, London, UK; 2 Department of Gastroenterology, North Tees and Hartlepool NHS Foundation Trust, Stockton-on-Tees, UK; 3 Department of Gastroenterology, Gloucestershire Hospitals NHS Foundation Trust, Cheltenham, UK

**Keywords:** COLORECTAL CANCER, SURVEILLANCE, POLYP

## Abstract

**Background:**

Recommendations for the first postpolypectomy surveillance colonoscopy (SC1), based on stratifying postpolypectomy colorectal cancer (CRC) risk, are well established. Limited data inform recommendations for surveillance beyond SC1.

**Objective:**

We investigated which patient groups need surveillance beyond SC1.

**Design:**

Retrospective analysis of patients who underwent colonoscopy with polypectomy at 17 UK hospitals, mostly from 2000 to 2010, and had ≥1 surveillance colonoscopies. Cancer and death data were collected through 2017. We examined patients in groups defined by risk at baseline and SC1, applying risk definitions from the 2020 UK postpolypectomy surveillance guidelines: ‘low risk, low risk’ (LR-LR), ‘high risk, low risk’ (HR-LR), ‘low risk, high risk’ (LR-HR) and ‘high risk, high risk’ (HR-HR). We examined CRC incidence after SC1, censoring at any second surveillance colonoscopy (SC2), and after SC2 through end of follow-up. We compared incidence with general population incidence using standardised incidence ratios (SIRs).

**Results:**

Analyses included 10 508 patients: LR-LR=6587 (63%), HR-LR=3272 (31%), LR-HR=248 (2%) and HR-HR=401 (4%). Median follow-up from SC1 was 8.0 years and 151 CRCs were diagnosed. Compared with the general population, CRC incidence after SC1 was lower in the LR-LR group (SIR 0.48, 95% CI 0.34 to 0.67), non-significantly different in the HR-LR (SIR 1.17, 0.85 to 1.58) or LR-HR (SIR 2.51, 0.81 to 5.85) groups, but higher in the HR-HR group (SIR 2.84, 1.30 to 5.39). After SC2, CRC incidence in the HR-HR group was no longer higher than in the general population (SIR 1.86, 0.89 to 3.42).

**Conclusion:**

Patients with high-risk findings at both baseline and SC1 needed an SC2, while those with low-risk findings at SC1 did not, regardless of their baseline findings.

**WHAT IS ALREADY KNOWN ON THIS TOPIC**

- Patients deemed to remain at increased risk of colorectal cancer (CRC) following polypectomy are recommended to undergo colonoscopy surveillance, and recommendations for the first surveillance colonoscopy (SC1), according to baseline polyp characteristics, are well established. This study was needed because few data exist to inform recommendations for surveillance beyond SC1.

**WHAT THIS STUDY ADDS**

- This study aimed to identify patient groups who remain at increased risk of CRC after SC1, compared with the general population, and so need ongoing surveillance. Examining CRC incidence after SC1 among 10 508 patients, with a median of 8 years’ follow-up, we found that patients with high-risk findings at both baseline and SC1 needed a second surveillance visit, while those with low-risk findings at SC1 did not, regardless of their baseline findings. We were unable to draw conclusions for those with low-risk findings at baseline and high-risk findings at SC1, although this combination of findings appears to be uncommon.

**HOW THIS STUDY MIGHT AFFECT RESEARCH, PRACTICE OR POLICY**

- Our data suggest that the classification of patients using polyp findings from SC1 alone may be sufficient to identify those needing a second surveillance visit, although this warrants further research.

## Introduction

 Patients deemed to remain at increased risk of colorectal cancer (CRC) following polypectomy are recommended to have colonoscopy surveillance. Recommendations for the first surveillance colonoscopy (SC1) according to baseline polyp characteristics are well established.^1^,^3^ Few data exist to inform recommendations for surveillance beyond SC1, acknowledged in the 2020 UK, European and US postpolypectomy surveillance guidelines.^1^,^3^ These guidelines differ in their recommendations for ongoing surveillance; for example, recommendations for the second surveillance colonoscopy (SC2) in the UK guidelines are based on SC1 findings only,^1^ while the European and US guidelines consider both baseline and SC1 findings.^2 3^ Surveillance is recommended to cease after one negative surveillance colonoscopy in the UK guidelines,^1^ but not until two negative surveillance colonoscopies in the European guidelines,^2^ while the US guidelines lack recommendations on stopping surveillance due to insufficient evidence.^3^

Studies have examined the likelihood of detecting advanced colorectal neoplasia at SC2 based on baseline and SC1 findings.^4^,^11^ To our knowledge, only one study has examined the influence of baseline and SC1 findings on long-term CRC outcomes.^12^ This study, which involved a retrospective cohort of patients who had adenomas removed at colonoscopy (the ‘All Adenomas’ cohort), identified patient, procedural and polyp characteristics at baseline and SC1 that were independently associated with increased CRC incidence. More recent analyses of the ‘All Adenomas’ cohort examined CRC incidence after SC1 in patient groups defined by baseline findings only.^13 14^

This present study extended the scope of these previous analyses to examine CRC incidence after SC1 in patient groups defined by baseline and SC1 findings. Our aim was to identify which patient groups need ongoing surveillance and when they could likely cease surveillance.

## Methods

We analysed data from the ‘All Adenomas’ cohort, which included patients who underwent colonoscopy with polypectomy at 17 UK hospitals from 1984 to 2010 (mostly (87%) from 2000 to 2010).^12 13 15^ The cohort was identified by searching endoscopy databases of participating hospitals for records of patients with colonic examinations before 2011. Hospital pathology databases were searched for records of colorectal lesions. Endoscopy and pathology records were matched and entered into a database.

Patient records were examined to identify the first adenoma sighting, defined as ‘baseline’. The ‘baseline visit’ could include a single examination or a series of examinations performed to completely examine the colon and remove detected polyps. Bowel preparation quality was graded by endoscopists as excellent, good, satisfactory or poor; when not graded by endoscopists, we classified bowel preparation quality as missing. Colonoscopies that reached the caecum, as recorded by endoscopists, or at which polyps were found in the caecum, were classified as complete examinations. Data were collected from the hospitals on colonic examinations performed through 2016; we grouped examinations occurring after the baseline visit into surveillance visits, using rules described elsewhere.^12^ Visits during which CRC was diagnosed were not counted as surveillance because they could not offer protection against CRC.

Patients who had a colonoscopy and ≥1 adenomas at baseline were assessed for eligibility. Patients who had CRC or colorectal resection before or at baseline, a condition associated with increased CRC risk (eg, inflammatory bowel disease, colitis, hamartomatous polyps, juvenile polyps, polyposis, a family history of familial adenomatous polyposis or Lynch syndrome), colorectal carcinoma in situ (now reported as high-grade dysplasia) in cancer registry data >3 years before baseline or a colonic examination for which the date was unknown were excluded from the cohort.

For the present analysis, we additionally excluded patients whose baseline visit was of suboptimal quality, defined as those where the most complete colonoscopy was either incomplete or of unknown completeness or the best bowel preparation was poor, considering the emphasis on high-quality baseline colonoscopies in postpolypectomy surveillance guidelines.^1^,^3^ Baseline bowel preparation quality was missing for 40% of the ‘All Adenomas’ cohort,^16^ due to the use of retrospective, routinely collected hospital data. We did not exclude these patients; we assumed that endoscopists would likely document poor bowel preparation, so when bowel preparation quality was missing, we assumed it was not poor. We excluded patients who did not attend surveillance within ≤6.5 years postbaseline; for patients whose first visit during follow-up was >6.5 years postbaseline, we deemed it more likely that they had returned for symptom investigation. This cut-off was chosen considering the longest recommended surveillance interval (5 years) in the 2002 UK guidelines,^17^ which governed surveillance for most of the study period, and allowed for delays. We excluded patients who, at SC1, were reported to have CRC, colorectal resection or a condition associated with increased CRC risk as detailed above. We excluded patients whom we could not classify into risk groups (described below) due to missing polyp information.

We classified patients into four groups based on the combinations of low-risk and/or high-risk findings at baseline and SC1. Following the 2020 UK postpolypectomy surveillance guidelines,^1^ high-risk findings included any of the following: ≥2 premalignant polyps (PMPs), of which ≥1 was ‘advanced’ (adenoma ≥10 mm or with high-grade dysplasia, or serrated polyp ≥10 mm or with dysplasia); ≥5 PMPs or ≥1 large (≥20 mm) non-pedunculated PMP. All other findings were considered low-risk findings.

Patients in the ‘low risk, low risk’ (LR-LR) group had low-risk findings at both baseline and SC1; those in the ‘high risk, low risk’ (HR-LR) group had high-risk findings at baseline and low-risk findings at SC1; those in the ‘low risk, high risk’ (LR-HR) group had low-risk findings at baseline and high-risk findings at SC1 and those in the ‘high risk, high risk’ (HR-HR) group had high-risk findings at both baseline and SC1.

Cancer registration and death certificate data were obtained from the National Health Service (NHS) Central Register, NHS England (formerly NHS Digital) and National Services Scotland through 2016 (Scotland) or 2017 (England). The primary outcome was incident CRC, defined as colorectal adenocarcinoma. Cancers with unspecified morphology located between the rectum and caecum were assumed to be adenocarcinomas^18^; those located anally were more likely to be squamous cell carcinomas and were not included as CRCs.

Exposures included the number of surveillance visits, sex, family history of cancer/CRC, age at SC1, year of SC1, bowel preparation quality at SC1 and examination completeness at SC1. We additionally examined the following polyp characteristics at both baseline and SC1: number of PMPs, number of advanced PMPs, PMP size, adenoma histology, adenoma dysplasia and presence of proximal PMPs.

To assess the risk of selection bias due to excluding patients who did not attend surveillance or could not be classified into one of the risk groups due to missing polyp information, the following baseline characteristics were examined: presence of low-risk or high-risk findings, sex, family history of cancer/CRC, age, year of baseline visit, bowel preparation quality and the previously mentioned polyp characteristics.

Detection and classification of serrated polyps were limited in the study era. Therefore, we did not create variables for serrated polyp characteristics, although we did use available serrated polyp data in our count of PMPs. We classified sessile serrated lesions and hyperplastic polyps as serrated polyps. We classified serrated adenomas and mixed hyperplastic-adenomatous polyps as adenomas, as they were classified in the study era, although noting that they are counted as serrated polyps in the 2020 UK surveillance guidelines.^1^

### Statistical analysis

We compared baseline characteristics between those included in the analysis and those who were excluded because they did not attend surveillance or could not be classified into a risk group using χ^2^ tests.

We compared sex, family history of cancer/CRC, age at SC1, year of SC1, bowel preparation quality at SC1, examination completeness at SC1 and number of surveillance visits between the four groups using χ^2^ tests. We used χ^2^ tests to compare baseline polyp characteristics between the LR-LR and LR-HR groups and between the HR-LR and HR-HR groups, and SC1 polyp characteristics between the LR-LR and HR-LR groups and between the LR-HR and HR-HR groups.

We examined CRC incidence after SC1. Time-at-risk started from the latest examination during SC1. End of follow-up was defined according to the date through which cancer and death data were available. Time-to-event data were censored at first diagnosis of CRC, emigration, death or end of follow-up, whichever occurred first. We included only the first diagnosed CRC per patient. We divided each patient’s follow-up time into two periods: after SC1, censoring at any second surveillance visit, and after SC2 through the date of final censoring.

We estimated cumulative CRC incidence with 95% CIs at 3 and 5 years after SC1 and showed time to CRC diagnosis using the Kaplan–Meier method. We compared CRC incidence with that in the general population using standardised incidence ratios (SIRs). We calculated SIRs, with exact Poisson 95% CIs, by dividing the observed by expected number of CRC cases, estimating expected cases by multiplying sex- and 5-year age-group-specific person years with corresponding CRC incidence rates in the general population of England in 2007 (approximately the midpoint of the follow-up period).^19^

We examined detection rates, with exact binomial 95% CIs, of advanced PMPs at SC2.

Our first sensitivity analysis classified patients into risk groups according to the 2020 European postpolypectomy surveillance guidelines,^2^ which define high-risk findings as ≥1 advanced PMPs or ≥5 adenomas. In our second sensitivity analysis, we excluded CRCs deemed likely to have developed from incompletely excised baseline PMPs: those found in the same or adjacent colorectal segment to PMPs that were ≥15 mm at baseline and sighted on ≥2 occasions within 5 years prior to CRC diagnosis, in line with previous methodology.^12^,^1520^ Additionally, when defining findings at SC1 and SC2, we excluded PMPs seen at surveillance that were deemed likely to have been incompletely excised at baseline: those found in the same or adjacent segment to a baseline PMP ≥15 mm that was sighted on ≥2 occasions within the preceding 3 years.^12 20^ These exclusions potentially made the data more representative of current practice considering recent improvements in polypectomy.^21^ In the third sensitivity analysis, we excluded patients whose baseline visit occurred before 2000, prior to the introduction of colonoscopy quality improvement initiatives in the UK.^22^ The fourth sensitivity analysis classified patients into groups based on SC1 findings only: everyone with low-risk findings at SC1 was in the ‘SC1 LR’ group and everyone with high-risk findings at SC1 was in the ‘SC1 HR’ group.

The ‘All Adenomas’ study is registered (ISRCTN15213649) and the protocol is online.^23^ We performed analyses in Stata/IC V.17 (StataCorp. 2021. Stata Statistical Software: Release 17. College Station, Texas: StataCorp LLC) and used a significance level of 0.05.

## Results

**Figure 1:**
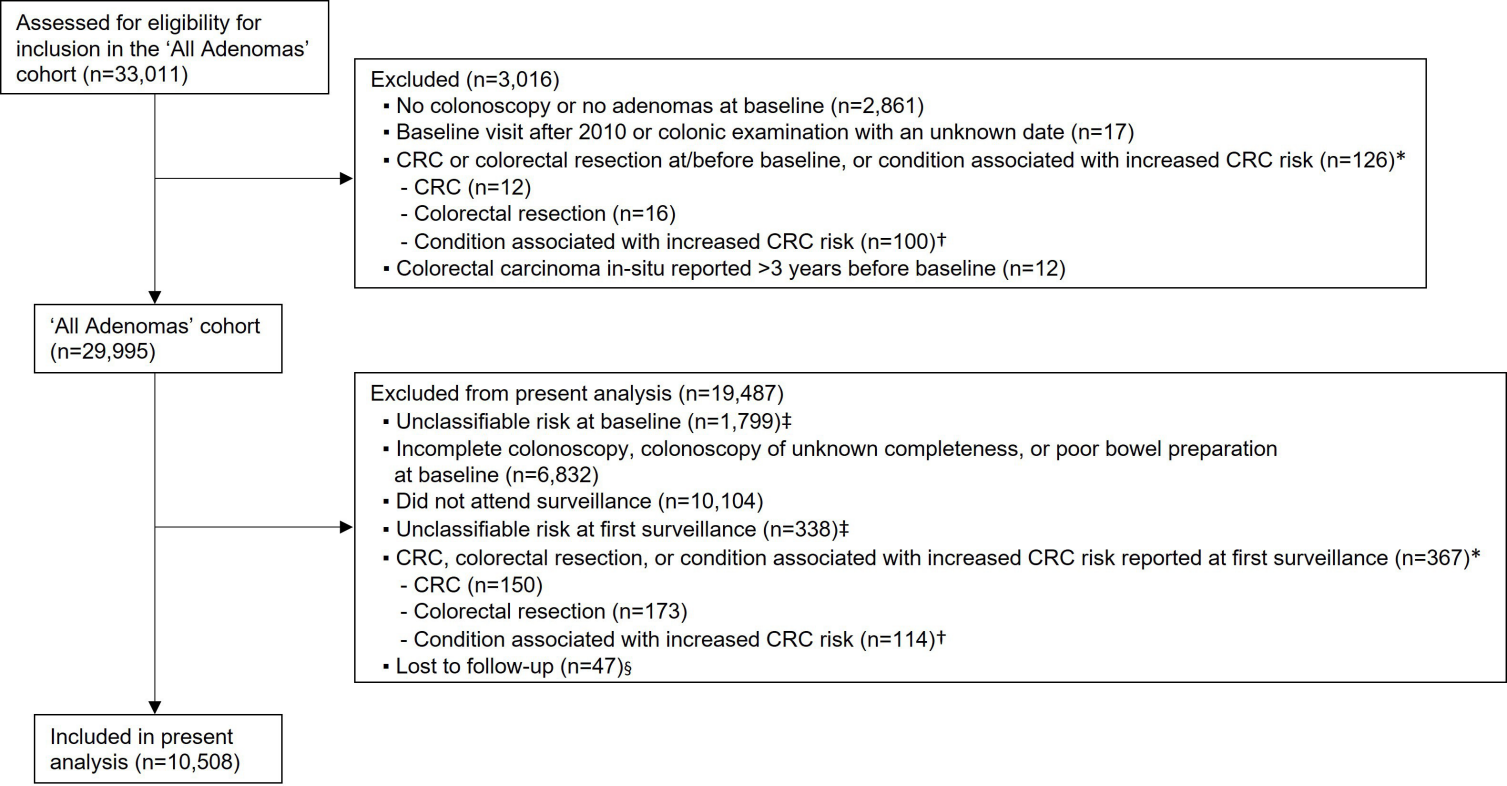
Flow diagram of the selection of the study population. *Non-mutually exclusive. †Including inflammatory bowel disease, colitis, hamartomatous polyps, juvenile polyps, polyposis, a family history of familial adenomatous polyposis and Lynch syndrome. ‡Not possible to classify findings at baseline or first surveillance as low risk or high risk due to missing information on polyp characteristics. §Reasons: had no surveillance and could not be traced through national data sources, had only one surveillance visit and could not be traced through national data sources beyond the visit, had all their examinations after emigrating or had an unknown date of birth. CRC, colorectal cancer.

**Figure 2:**
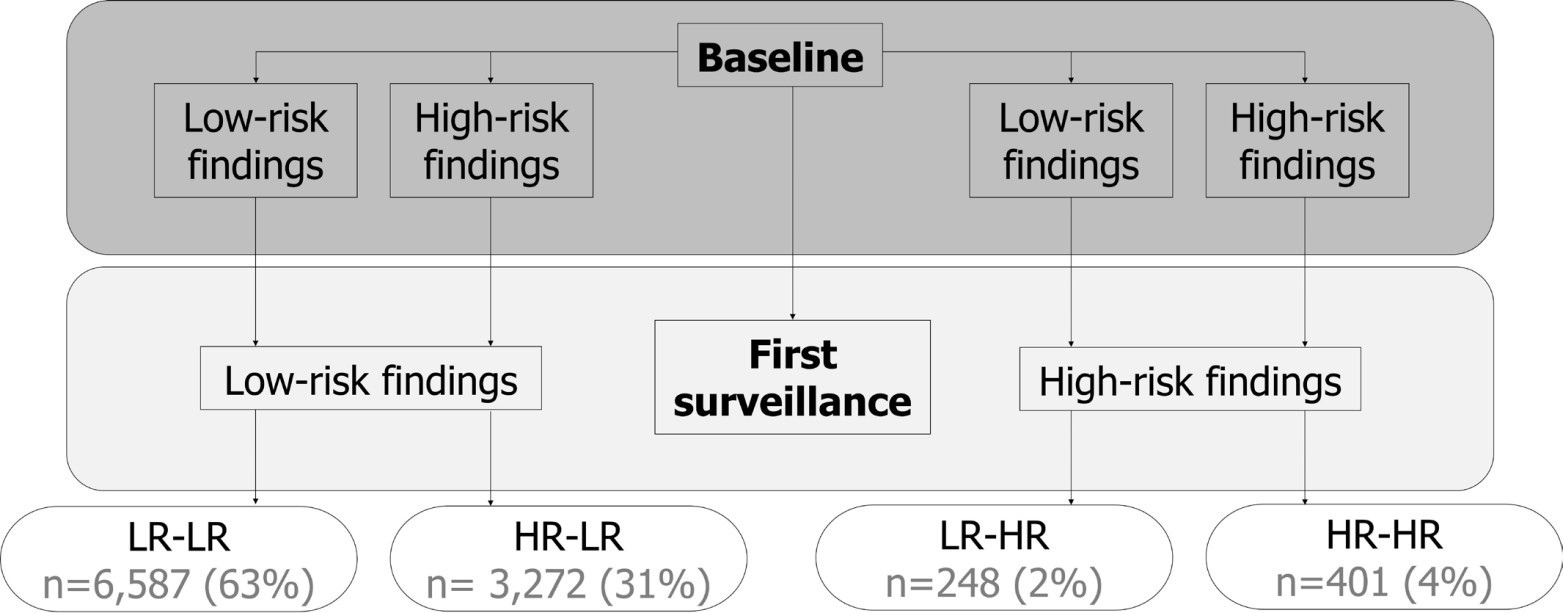
Classification of risk groups according to findings at baseline and first surveillance (n=10 508)*.*Patients in the LR-LR group had low-risk findings at both baseline and first surveillance; those in the HR-LR group had high-risk findings at baseline and low-risk findings at first surveillance; those in the LR-HR group had low-risk findings at baseline and high-risk findings at first surveillance and those in the HR-HR group had high-risk findings at both baseline and first surveillance. High-risk findings were defined as ≥2 PMPs of which ≥1 was ‘advanced’ (adenoma ≥10 mm or with high-grade dysplasia, or serrated polyp ≥10 mm or with any dysplasia), ≥5 PMPs or ≥1 large (≥20 mm) non-pedunculated PMP; findings not meeting these criteria were considered low-risk findings. HR-HR, high risk, high risk; HR-LR, high risk, low risk; LR-HR, low risk, high risk; LR-LR, low risk, low risk; PMP, premalignant polyp.

Of 33 011 patients assessed for eligibility for the ‘All Adenomas’ cohort, we excluded 2861 with no colonoscopy or adenomas at baseline; 17 with a baseline visit after 2010 or colonic examination with an unknown date; 126 with CRC or colorectal resection before or at baseline or a condition associated with increased CRC risk and 12 with colorectal carcinoma in situ >3 years before baseline. For the present analysis, we additionally excluded 1799 patients who were missing information needed to classify their baseline findings as low or high risk; 6832 with a suboptimal quality baseline colonoscopy; 10 104 with no surveillance; 338 who were missing information needed to classify their SC1 findings as low or high risk; 367 with CRC, colorectal resection or a condition associated with increased CRC risk at SC1 and 47 who were lost to follow-up (figure 1). This left 10 508 patients for analysis, of whom 6587 (63%) were in the LR-LR group, 3272 (31%) in the HR-LR group, 248 (2%) in the LR-HR group and 401 (4%) in the HR-HR group (figure 2).

Comparing baseline characteristics between those included in the analysis and those who were excluded because they did not attend surveillance or could not be classified into a risk group (n=11 387), excluded patients were generally more likely to have baseline characteristics typically associated with lower CRC risk (online supplemental table 1).

Greater proportions of the LR-LR and LR-HR groups were women, reported a family history of cancer/CRC, were aged <65 years at SC1 and had their SC1 in 2011–2017, compared with the HR-LR and HR-HR groups. At SC1, smaller proportions of the LR-LR and HR-LR groups had excellent or good bowel preparation quality, and greater proportions had an incomplete colonoscopy or no confirmed colonoscopy, compared with the LR-HR and HR-HR groups. The number of patients in each group who attended a single surveillance visit was 3509 (53%) in the LR-LR group, 1406 (43%) in the HR-LR group, 84 (34%) in the LR-HR group and 119 (30%) in the HR-HR group; remaining patients attended ≥2 surveillance visits. In the LR-LR, HR-LR, LR-HR and HR-HR groups, respectively, median intervals from baseline to SC1 were 3.0 (IQR, 2.0 to 4.0), 2.1 (1.1 to 3.2), 3.1 (2.3 to 4.4) and 1.8 (1.1 to 3.2) years, and median intervals from SC1 to SC2 were 3.1 (2.4 to 4.6), 3.0 (2.2 to 3.4), 2.4 (1.3 to 3.3) and 1.8 (1.1 to 3.0) years (table 1).

**Table 1:** Characteristics of patients, surveillance visits and polyps seen at baseline and first surveillance, by risk group (n=10 508)

Characteristic	Risk group*			
	LR-LR n (%)	HR-LR n (%)	LR-HR n (%)	HR-HR n (%)
Total	6587 (100.0)	3272 (100.0)	248 (100.0)	401 (100.0)
Sex†				
Women	2950 (44.8)	1144 (35.0)	90 (36.3)	126 (31.4)
Men	3637 (55.2)	2128 (65.0)	158 (63.7)	275 (68.6)
Family history of cancer/CRC†‡				
No	5736 (87.1)	3085 (94.3)	219 (88.3)	383 (95.5)
Yes	851 (12.9)	187 (5.7)	29 (11.7)	18 (4.5)
Age at first surveillance visit, years†				
<55	1327 (20.1)	370 (11.3)	35 (14.1)	29 (7.2)
55–64	1864 (28.3)	877 (26.8)	76 (30.6)	108 (26.9)
65–74	2269 (34.4)	1404 (42.9)	89 (35.9)	176 (43.9)
≥75	1127 (17.1)	621 (19.0)	48 (19.4)	88 (21.9)
Year of first surveillance visit†				
1984–1999	376 (5.7)	200 (6.1)	12 (4.8)	21 (5.2)
2000–2004	1176 (17.9)	585 (17.9)	31 (12.5)	76 (19.0)
2005–2010	3365 (51.1)	1865 (57.0)	147 (59.3)	235 (58.6)
2011–2017	1670 (25.4)	622 (19.0)	58 (23.4)	69 (17.2)
Bowel preparation quality at first surveillance visit†				
Excellent or good	1684 (25.6)	928 (28.4)	83 (33.5)	132 (32.9)
Satisfactory	1037 (15.7)	518 (15.8)	43 (17.3)	64 (16.0)
Poor	338 (5.1)	160 (4.9)	7 (2.8)	23 (5.7)
Missing	2889 (43.9)	1393 (42.6)	108 (43.5)	161 (40.1)
No known colonoscopy	639 (9.7)	273 (8.3)	7 (2.8)	21 (5.2)
Examination completeness at first surveillance visit†				
Complete colonoscopy	5120 (77.7)	2630 (80.4)	221 (89.1)	335 (83.5)
Incomplete colonoscopy	315 (4.8)	145 (4.4)	2 (0.8)	11 (2.7)
Colonoscopy of unknown completeness	513 (7.8)	224 (6.8)	18 (7.3)	34 (8.5)
No known colonoscopy	639 (9.7)	273 (8.3)	7 (2.8)	21 (5.2)
No. of surveillance visits†§				
1	3509 (53.3)	1406 (43.0)	84 (33.9)	119 (29.7)
2	2014 (30.6)	1073 (32.8)	101 (40.7)	126 (31.4)
≥3	1064 (16.2)	793 (24.2)	63 (25.4)	156 (38.9)

	Risk group*							
	LR-LR n (%)		HR-LR n (%)		LR-HR n (%)		HR-HR n (%)	
	Baseline¶	First surveillance**	Baseline¶	First surveillance**	Baseline¶	First surveillance**	Baseline¶	First surveillance**
No. of PMPs								
0	0 (0.0)	4741 (72.0)	0 (0.0)	1914 (58.5)	0 (0.0)	0 (0.0)	0 (0.0)	0 (0.0)
1	5114 (77.6)	1323 (20.1)	241 (7.4)	840 (25.7)	163 (65.7)	20 (8.1)	36 (9.0)	48 (12.0)
2	924 (14.0)	354 (5.4)	1305 (39.9)	314 (9.6)	46 (18.5)	79 (31.9)	115 (28.7)	118 (29.4)
3	387 (5.9)	129 (2.0)	658 (20.1)	149 (4.6)	25 (10.1)	33 (13.3)	58 (14.5)	59 (14.7)
4	162 (2.5)	40 (0.6)	324 (9.9)	55 (1.7)	14 (5.6)	29 (11.7)	51 (12.7)	46 (11.5)
≥5	0 (0.0)	0 (0.0)	744 (22.7)	0 (0.0)	0 (0.0)	87 (35.1)	141 (35.2)	130 (32.4)
No. of advanced PMPs								
0	4346 (66.0)	6381 (96.9)	237 (7.2)	3096 (94.6)	169 (68.1)	66 (26.6)	31 (7.7)	81 (20.2)
1	2241 (34.0)	206 (3.1)	2052 (62.7)	176 (5.4)	79 (31.9)	144 (58.1)	203 (50.6)	249 (62.1)
2	0 (0.0)	0 (0.0)	751 (23.0)	0 (0.0)	0 (0.0)	32 (12.9)	110 (27.4)	57 (14.2)
≥3	0 (0.0)	0 (0.0)	232 (7.1)	0 (0.0)	0 (0.0)	6 (2.4)	57 (14.2)	14 (3.5)
PMP size, mm††								
<10	4419 (67.1)	1651 (89.4)	296 (9.0)	1191 (87.7)	172 (69.4)	65 (26.2)	35 (8.7)	87 (21.7)
10–19	1502 (22.8)	169 (9.2)	1601 (48.9)	147 (10.8)	50 (20.2)	127 (51.2)	172 (42.9)	197 (49.1)
≥20	651 (9.9)	23 (1.2)	1358 (41.5)	18 (1.3)	25 (10.1)	49 (19.8)	192 (47.9)	111 (27.7)
Unknown	15 (0.2)	3 (0.2)	17 (0.5)	2 (0.1)	1 (0.4)	7 (2.8)	2 (0.5)	6 (1.5)
Adenoma histology‡‡								
Tubular	4282 (65.0)	1156 (76.7)	1255 (38.4)	831 (75.8)	156 (62.9)	115 (50.4)	132 (32.9)	172 (46.7)
Tubulovillous	1735 (26.3)	216 (14.3)	1576 (48.2)	181 (16.5)	64 (25.8)	87 (38.2)	193 (48.1)	129 (35.1)
Villous	189 (2.9)	24 (1.6)	352 (10.8)	23 (2.1)	13 (5.2)	13 (5.7)	58 (14.5)	44 (12.0)
Unknown	381 (5.8)	112 (7.4)	89 (2.7)	62 (5.7)	15 (6.0)	13 (5.7)	18 (4.5)	23 (6.3)
Adenoma dysplasia§§								
Low grade	5951 (90.3)	1389 (92.1)	2437 (74.5)	1030 (93.9)	220 (88.7)	200 (87.7)	288 (71.8)	296 (80.4)
High grade	421 (6.4)	34 (2.3)	761 (23.3)	24 (2.2)	14 (5.6)	19 (8.3)	100 (24.9)	42 (11.4)
Unknown	215 (3.3)	85 (5.6)	74 (2.3)	43 (3.9)	14 (5.6)	9 (3.9)	13 (3.2)	30 (8.2)
Proximal PMPs¶¶								
No	4042 (61.4)	5610 (85.2)	1305 (39.9)	2485 (75.9)	123 (49.6)	66 (26.6)	120 (29.9)	139 (34.7)
Yes	2545 (38.6)	977 (14.8)	1967 (60.1)	787 (24.1)	125 (50.4)	182 (73.4)	281 (70.1)	262 (65.3)

* Patients in the LR-LR group had low-risk findings at both baseline and first surveillance; those in the HR-LR group had high-risk findings at baseline and low-risk findings at first surveillance; those in the LR-HR group had low-risk findings at baseline and high-risk findings at first surveillance and those in the HR-HR group had high-risk findings at both baseline and first surveillance. High-risk findings were defined as ≥2 PMPs of which ≥1 was ‘advanced’ (adenoma ≥10 mm or with high-grade dysplasia, or serrated polyp ≥10 mm or with any dysplasia), ≥5 PMPs or ≥1 large (≥20 mm) non-pedunculated PMP; findings not meeting these criteria were considered low-risk findings.

† Comparing these characteristics between the four groups, using the χ2 test, all comparisons had p values ≤0.05.

‡ Family history of cancer/CRC was defined as family history of cancer or CRC recorded by the patient’s endoscopist at an examination before or during the baseline visit. Of 328 patients reported to have a family history of cancer, 207 (63%) were from a specialist hospital for colorectal diseases, so it was reasonable to assume that they had a family history of CRC. For the remaining 121 patients, it was not possible to determine whether they had a family history of CRC or a family history of other types of cancer.

§ In the LR-LR, HR-LR, LR-HR and HR-HR groups, respectively, the median interval from baseline to first surveillance was 3.0 (IQR, 2.0 to 4.0), 2.1 (1.1 to 3.2), 3.1 (2.3 to 4.4) and 1.8 (1.1 to 3.2) years; the median interval from first to second surveillance was 3.1 (2.4 to 4.6), 3.0 (2.2 to 3.4), 2.4 (1.3 to 3.3) and 1.8 (1.1 to 3.0) years and the median follow-up time from first surveillance through the date of final censoring was 8.0 (5.6 to 11.0), 8.0 (5.7 to 10.8), 7.1 (4.9 to 9.8) and 8.0 (5.4 to 10.6) years.

¶ Comparing baseline polyp characteristics between the LR-LR and LR-HR groups, using the χ2 test, the following comparisons had p values ≤0.05: number of PMPs and the presence of proximal PMPs. Comparing baseline polyp characteristics between the HR-LR and HR-HR groups, using the χ2 test, the following comparisons had p values ≤0.05: number of PMPs, number of advanced PMPs, adenoma histology and the presence of proximal PMPs.

** Comparing polyp characteristics at first surveillance between the LR-LR and HR-LR groups, using the χ2 test, the following comparisons had p values ≤0.05: number of PMPs, number of advanced PMPs and the presence of proximal PMPs. Comparing polyp characteristics at first surveillance between the LR-HR and HR-HR groups, using the χ2 test, the following comparisons had p values ≤0.05: adenoma dysplasia and the presence of proximal PMPs.

†† Defined according to the largest PMP recorded during the baseline (or first surveillance) visit.

‡‡ Defined according to the greatest degree of villousness recorded during the baseline (or first surveillance) visit; percentages were calculated using the number of patients with at least one adenoma as the denominator.

§§ Defined according to the highest grade of dysplasia recorded during the baseline (or first surveillance) visit; percentages were calculated using the number of patients with at least one adenoma as the denominator.

¶¶ Proximal was defined as proximal to the descending colon.

CRC, colorectal cancer; HR-HR, high risk, high risk; HR-LR, high risk, low risk; LR-HR, low risk, high risk; LR-LR, low risk, low risk; PMP, premalignant polyp.

Compared with the LR-LR group, a greater proportion of the LR-HR group had ≥2 PMPs or proximal PMPs at baseline. Compared with the HR-LR group, a greater proportion of the HR-HR group had ≥4 PMPs, ≥2 advanced PMPs, adenomas with villous or unknown histology or proximal PMPs at baseline (table 1). Compared with the LR-LR group, the HR-LR group was more likely to have had ≥1 PMPs, an advanced PMP or proximal polyps at SC1. Compared with the LR-HR group, the HR-HR group was more likely to have had an adenoma with high-grade dysplasia but less likely to have had proximal polyps at SC1 (table 1).

Overall, median follow-up from SC1 was 8.0 years (IQR 5.6–10.9), during which 60, 66, 6 and 19 patients in the LR-LR, HR-LR, LR-HR and HR-HR groups, respectively, were diagnosed with CRC (table 2). After SC1, in the presence of SC1 only, cumulative CRC incidence at 3 years was 0.2% (95% CI 0.1 to 0.4) in the LR-LR group, 0.5% (0.3 to 0.8) in the HR-LR group, not estimable in the LR-HR group because there were no CRC cases, and 1.7% (0.6 to 4.8) in the HR-HR group. At 5 years after SC1, corresponding figures were 0.3% (95% CI 0.2 to 0.5), 1.5% (1.0 to 2.2), 4.0% (1.3 to 12.0) and 4.2% (1.7 to 10.3) in the LR-LR, HR-LR, LR-HR and HR-HR groups, respectively (table 2).

**Table 2:** Incidence of CRC and age–sex SIRs, by risk group (n=10 508)

Risk group*	No. of patients, n (%)	Person years	Total no. of CRC cases	Incidence rate per 100 000 person years (95% CI)	At 3 years†		At 5 years†		Standardisation	
					No. of cases	Cumulative incidence, % (95% CI)‡	No. of cases	Cumulative incidence, % (95% CI)‡	No. of expected cases	SIR (95% CI)
After the first surveillance visit, censored at any second surveillance visit§										
LR-LR	6587 (62.7)	35 337	35	99 (71 to 138)	14	0.2 (0.1 to 0.4)	17¶	0.3 (0.2 to 0.5)	72	0.48 (0.34 to 0.67)
HR-LR	3272 (31.1)	15 214	43	283 (210 to 381)	13	0.5 (0.3 to 0.8)	28¶	1.5 (1.0 to 2.2)	37	1.17 (0.85 to 1.58)
LR-HR	248 (2.4)	906	5	552 (230 to 1326)	0	‘’	3¶	4.0 (1.3 to 12.0)	2	2.51 (0.81 to 5.85)
HR-HR	401 (3.8)	1236	9	728 (379 to 1399)	4	1.7 (0.6 to 4.8)	6¶	4.2 (1.7 to 10.3)	3	2.84 (1.30 to 5.39)
After the second surveillance visit through the date of final censoring**										
LR-LR	3078 (57.1)	20 674	25	121 (82 to 179)	3	0.1 (0.0 to 0.3)	9††	0.4 (0.2 to 0.8)	45	0.56 (0.36 to 0.82)
HR-LR	1866 (34.6)	12 328	23	187 (124 to 281)	5	0.3 (0.1 to 0.7)	10††	0.7 (0.4 to 1.2)	32	0.72 (0.46 to 1.09)
LR-HR	164 (3.0)	954	1	105 (15 to 744)	0	‘’	0††	‘’	2	0.43 (0.01 to 2.38)
HR-HR	282 (5.2)	1986	10	503 (271 to 936)	1	0.4 (0.1 to 2.9)	4††	1.8 (0.7 to 4.7)	5	1.86 (0.89 to 3.42)

* Patients in the LR-LR group had low-risk findings at both baseline and first surveillance; those in the HR-LR group had high-risk findings at baseline and low-risk findings at first surveillance; those in the LR-HR group had low-risk findings at baseline and high-risk findings at first surveillance and those in the HR-HR group had high-risk findings at both baseline and first surveillance. High-risk findings were defined as ≥2 PMPs of which ≥1 was ‘advanced’ (adenoma ≥10 mm or with high-grade dysplasia, or serrated polyp ≥10 mm or with any dysplasia), ≥5 PMPs or ≥1 large (≥20 mm) non-pedunculated PMP; findings not meeting these criteria were considered low-risk findings.

† For analyses of incidence after first surveillance, cumulative incidence data are shown for 3 and 5 years after first surveillance. For analyses of incidence after second surveillance, cumulative incidence data are shown for 3 and 5 years after second surveillance.

‡ Estimated using the Kaplan–Meier method.

§ Each patient’s follow-up time was included from their first surveillance visit and censored at any second surveillance visit.

¶ The remaining CRC cases were diagnosed during the follow-up period starting 5 years after first surveillance (LR-LR, n=18; HR-LR, n=15; LR-HR, n=2 and HR-HR, n=3).

** For those who attended ≥2 surveillance visits, each patient’s follow-up time was included from their second surveillance visit through the date of final censoring.

†† The remaining CRC cases were diagnosed during the follow-up period starting 5 years after second surveillance (LR-LR, n=16; HR-LR, n=13; LR-HR, n=1 and HR-HR, n=6).

CRC, colorectal cancer; HR-HR, high risk, high risk; HR-LR, high risk, low risk; LR-HR, low risk, high risk; LR-LR, low risk, low risk; PMP, premalignant polyp; SIR, standardised incidence ratio.

**Figure 3:**
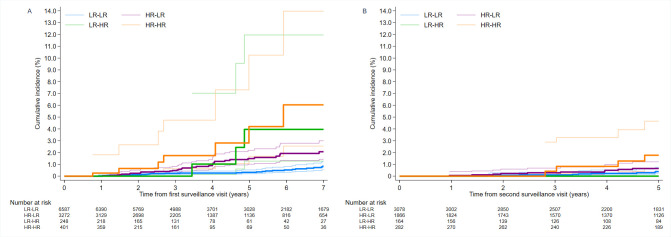
Kaplan–Meier estimates of cumulative CRC incidence (**A**) after first surveillance and (**B**) after second surveillance, by risk group (n=10 508)*. 95% CIs are shown for each curve, represented by the thin lines, while the cumulative CRC incidence point estimates are represented by the thicker lines. (**A**) Each patient’s follow-up time was included from their first surveillance visit and censored at any second surveillance visit. In total, 35, 43, 5 and 9 CRC cases from the LR-LR, HR-LR, LR-HR and HR-HR groups, respectively, were included in the counts for cumulative CRC incidence after first surveillance. (**B**) For those who attended ≥2 surveillance visits, each patient’s follow-up time was included from their second surveillance visit through the date of final censoring. In total, 25, 23, 1 and 10 CRC cases from the LR-LR, HR-LR, LR-HR and HR-HR groups, respectively, were included in the counts for cumulative CRC incidence after second surveillance. *Patients in the LR-LR group had low-risk findings at both baseline and first surveillance; those in the HR-LR group had high-risk findings at baseline and low-risk findings at first surveillance; those in the LR-HR group had low-risk findings at baseline and high-risk findings at first surveillance and those in the HR-HR group had high-risk findings at both baseline and first surveillance. High-risk findings were defined as ≥2 PMPs of which ≥1 was ‘advanced’ (adenoma ≥10 mm or with high-grade dysplasia, or serrated polyp ≥10 mm or with any dysplasia), ≥5 PMPs or ≥1 large (≥20 mm) non-pedunculated PMP; findings not meeting these criteria were considered low-risk findings. CRC, colorectal cancer; HR-HR, high risk, high risk; HR-LR, high risk, low risk; LR-HR, low risk, high risk; LR-LR, low risk, low risk; PMP, premalignant polyp.

After SC2, in the presence of SC2 and any subsequent surveillance visits, cumulative CRC incidence at 3 years was 0.1% (95% CI 0.0 to 0.3) in the LR-LR group, 0.3% (0.1 to 0.7) in the HR-LR group and 0.4% (0.1 to 2.9) in the HR-HR group. At 5 years after SC2, cumulative CRC incidence was 0.4% (95% CI 0.2 to 0.8) in the LR-LR group, 0.7% (0.4 to 1.2) in the HR-LR group and 1.8% (0.7 to 4.7) in the HR-HR group. At 3 and 5 years after SC2, cumulative CRC incidence was not estimable in the LR-HR group (table 2). Kaplan–Meier curves illustrating cumulative CRC incidence after SC1 and SC2 are shown in figure 3.

Compared with the general population, CRC incidence after SC1, in the presence of SC1 only, was lower in the LR-LR group (SIR 0.48, 95% CI 0.34 to 0.67), not significantly different in the HR-LR (SIR 1.17, 0.85 to 1.58) or LR-HR (SIR 2.51, 0.81 to 5.85) groups, but higher in the HR-HR group (SIR 2.84, 1.30 to 5.39). After SC2, in the presence of SC2 and any subsequent visits, CRC incidence was lower in the LR-LR group (SIR 0.56, 95% CI 0.36 to 0.82) and not significantly different in the HR-LR (SIR 0.72, 0.46 to 1.09), LR-HR (SIR 0.43, 0.01 to 2.38) or HR-HR (SIR 1.86, 0.89 to 3.42) groups, compared with the general population. The SIR 95% CIs for the LR-HR group were wide due to few CRC cases (table 2).

In the LR-LR, HR-LR, LR-HR and HR-HR groups, respectively, detection rates of advanced PMPs at SC2 were 5.4% (95% CI 4.6 to 6.3), 9.1% (7.8 to 10.5), 12.2% (7.6 to 18.2) and 17.4% (13.1 to 22.3) (table 3).

**Table 3:** Detection rates of advanced PMPs at second surveillance, by risk group (n=10 508)

Risk group*	Total no. of patients	No. of patients attending a second surveillance visit, n (%)	Interval from first to second surveillance in years, median (IQR)†	Detection rate of advanced PMPs at second surveillance, % (95% CI)‡
LR-LR	6587	3078 (46.7)	3.1 (2.4 to 4.6)	5.4 (4.6 to 6.3)
HR-LR	3272	1866 (57.0)	3.0 (2.2 to 3.4)	9.1 (7.8 to 10.5)
LR-HR	248	164 (66.1)	2.4 (1.3 to 3.3)	12.2 (7.6 to 18.2)
HR-HR	401	282 (70.3)	1.8 (1.1 to 3.0)	17.4 (13.1 to 22.3)

* Patients in the LR-LR group had low-risk findings at both baseline and first surveillance; those in the HR-LR group had high-risk findings at baseline and low-risk findings at first surveillance; those in the LR-HR group had low-risk findings at baseline and high-risk findings at first surveillance and those in the HR-HR group had high-risk findings at both baseline and first surveillance. High-risk findings were defined as ≥2 PMPs of which ≥1 was ‘advanced’ (adenoma ≥10 mm or with high-grade dysplasia, or serrated polyp ≥10 mm or with any dysplasia), ≥5 PMPs or ≥1 large (≥20 mm) non-pedunculated PMP; findings not meeting these criteria were considered low-risk findings.

† Of the 5390 patients who had a second surveillance visit, 5179 (96%) had their second surveillance visit within ≤6.5 years of their first surveillance visit. Excluding the 211 patients who had their second surveillance visit >6.5 years after their first surveillance visit (LR-LR, n=157; HR-LR, n=51; LR-HR, n=2 and HR-HR, n=1), detection rates of advanced PMPs at second surveillance were 5.1% (95% CI 4.4 to 6.0), 8.8% (7.6 to 10.2), 11.7% (7.2 to 17.7) and 17.4% (13.2 to 22.4) in the LR-LR, HR-LR, LR-HR and HR-HR groups, respectively.

‡ Advanced PMPs were defined as an adenoma ≥10 mm or with high-grade dysplasia, or a serrated polyp ≥10 mm or with any dysplasia.

HR-HR, high risk, high risk; HR-LR, high risk, low risk; LR-HR, low risk, high risk; LR-LR, low risk, low risk; PMP, premalignant polyp.

Applying the European rather than the UK surveillance guidelines, the number of included patients differed (10 328 compared with 10 508) due to differences in numbers of patients with unclassifiable risk by each guideline (data not shown). Of the 10 328 included patients, 4173 (40%), 5197 (50%), 250 (2%) and 708 (7%) were in the LR-LR, HR-LR, LR-HR and HR-HR groups, respectively. SIRs were slightly lower in the HR-LR, LR-HR and HR-HR groups, compared with the main analysis (online supplemental table 2).

In both the sensitivity analysis excluding incompletely excised baseline PMPs and the sensitivity analysis excluding patients whose baseline visit occurred before 2000, SIRs in the LR-LR, HR-LR and LR-LR groups were similar to those observed in the main analysis. In the HR-HR group, CRC incidence after SC1 was not significantly different to that in the general population (online supplemental tables 3 and 4), contrasting with the main analysis findings; however, there were only six CRC cases in the HR-HR group in both sensitivity analyses. Detection rates of advanced PMPs at SC2 in these sensitivity analyses were generally similar to those observed in the main analysis (online supplemental table 5).

Classifying patients by SC1 findings only, 9859 (94%) and 649 (6%) were in the ‘SC1 LR’ and ‘SC1 HR’ groups, respectively. Compared with the general population, CRC incidence after SC1 was lower in the ‘SC1 LR’ group (SIR 0.72, 95% CI 0.57 to 0.89) and higher in the ‘SC1 HR’ group (SIR 2.71, 1.48 to 4.55); after SC2, CRC incidence was no longer higher in the ‘SC1 HR’ group (SIR 1.43, 0.71 to 2.55) (online supplemental table 6). Detection rates of advanced PMPs at SC2 were 6.8% (95% CI 6.1 to 7.5) and 15.7% (12.4 to 19.5) in the ‘SC1 LR’ and ‘SC1 HR’ groups, respectively (online supplemental table 5).

## Discussion

This study examined CRC incidence over a median of 8 years after SC1 among 10 508 patients grouped by polyp findings at baseline and SC1, with 6587 (63%) in the LR-LR group, 3272 (31%) in the HR-LR group, 248 (2%) in the LR-HR group and 401 (4%) in the HR-HR group. Patients in the HR-HR group needed a second surveillance visit, those in the LR-LR and HR-LR groups did not, while we were unable to draw conclusions for the LR-HR group.

Among all patients, 6835 (65%) had low-risk findings and 3673 (35%) had high-risk findings at baseline. Most of those with low-risk findings at baseline had low-risk findings at SC1 (96%, n=6587); similarly, 89% (n=3272) of those with high-risk findings at baseline had low-risk findings at SC1. This shows that following a complete baseline colonoscopy with polypectomy, high-risk findings at SC1 were uncommon. Among patients with low-risk findings at baseline, high-risk findings at SC1 were more common among those with multiple or proximal PMPs at baseline, while among patients with high-risk findings at baseline, high-risk findings at SC1 were more common among those with greater numbers of PMPs, adenomas with villous histology or proximal PMPs at baseline. This likely reflects the increased risk of missing PMPs when multiple PMPs are present or incompletely excising advanced or proximal PMPs.^24 25^

After SC1, in the presence of SC1 only, CRC incidence was nearly three times higher in the HR-HR group, not significantly different in the HR-LR or LR-HR groups, and approximately 0.5 times lower in the LR-LR group, compared with the general population. The SIR 95% CI for the LR-HR group was wide due to few CRC cases, so it is plausible that we had insufficient power to detect a difference between this group and the general population. The finding that patients with high-risk findings at both baseline and SC1 were at increased CRC risk compared with the general population, possibly reflecting a propensity for further PMP development or missed or incompletely excised PMPs at baseline or SC1, shows that they needed a second surveillance visit. Whether patients with low-risk findings at baseline and high-risk findings at SC1 needed a second surveillance visit is unclear; however, this combination of findings was uncommon, occurring in 2% of our cohort. It is worth remembering that these patients would not have been offered a first surveillance visit under the 2020 UK or European surveillance guidelines.^1 2^ Patients with low-risk findings at SC1 did not need ongoing surveillance, regardless of their baseline findings, supporting the UK surveillance guideline recommendation for such patients to participate in non-invasive CRC screening when invited, rather than have ongoing surveillance.^1^

After SC2, in the presence of SC2 and any subsequent surveillance visits, the HR-HR group was no longer at increased CRC risk compared with the general population. We were unable to further stratify by the number of surveillance visits due to limited CRC cases, preventing understanding of whether the excess CRC risk in this group was reduced after SC2 or subsequent visits. Therefore, we could not determine whether a second surveillance visit alone was sufficient for this group or whether additional visits might have been needed.

Our data suggest that there may be limited value in considering both baseline and SC1 findings to identify patients needing ongoing surveillance. When we considered baseline findings in patients with low-risk findings at SC1, we identified a group at low CRC risk after SC1 (LR-LR) and another group whose CRC risk after SC1 was similar to that in the general population (HR-LR). In a resource-constrained setting like the UK, where surveillance is offered only to those at increased CRC risk compared with the general population, neither of these patient groups would be offered ongoing surveillance. When we considered baseline findings in patients with high-risk findings at SC1, we identified a group at increased CRC risk after SC1 (HR-HR) and another group with too few CRC cases to draw conclusions (LR-HR), although this combination of findings appears to be uncommon.

Previous studies investigated whether considering both baseline and SC1 findings is more useful than considering SC1 findings alone for assessing the likelihood of finding advanced adenomas (AAs: ≥10 mm, tubulovillous/villous histology or high-grade dysplasia) or high-risk findings (typically, ≥1 AA or ≥3 adenomas) at SC2.^5^,^11^ Two studies found that, for patients with low-risk or high-risk findings at SC1, baseline findings added no information about the likelihood of detecting high-risk findings at SC2.^6 7^ In others, baseline findings added information about the likelihood of detecting AAs or high-risk findings at SC2 for those with low-risk findings at SC1^59^,^11^ and/or for those with high-risk findings at SC1.^5 8 10^ These studies focused on statistical differences in detection rates between groups, although it is important to consider the clinical significance of any differences.

In our analysis, among patients with low-risk findings at SC1, advanced PMP detection rates at SC2 were nearly two times as high in those with high-risk findings (9%) compared with those with low-risk findings (5%) at baseline. The differences are unlikely to be clinically important because detection rates in both groups were below the minimum threshold for advanced PMP yield to justify surveillance (10%) suggested in the UK surveillance guidelines.^1^ Among patients with high-risk findings at SC1, advanced PMP detection rates at SC2 were above this threshold in those with low-risk findings at baseline (12%) and those with high-risk findings at baseline (17%). Again, this suggests that considering baseline findings in addition to SC1 findings does not add useful information for decisions regarding ongoing surveillance.

Applying the European postpolypectomy surveillance guidelines,^2^ CRC incidence estimates were slightly lower in the HR-LR, LR-HR and HR-HR groups than in the main analysis. This might reflect the movement of patients with a single advanced PMP (a low-risk finding in the UK guidelines but high-risk finding in the European guidelines) from the LR-LR group into the other groups. Our conclusions remained the same, indicating that our findings may be applicable to surveillance settings following the UK or European guidelines.

When we excluded CRCs likely to have arisen from incompletely excised baseline PMPs, as well as PMPs seen at surveillance that were likely to have been incompletely excised at baseline, CRC incidence after SC1 was not significantly different in the HR-HR group, compared with the general population, contrasting with the main analysis findings. This was also observed when excluding patients whose baseline visit occurred before 2000. It is difficult to determine whether these findings reflect a true lack of difference in CRC incidence between the HR-HR group and the general population or whether there was insufficient power to detect a difference, as there were only six CRC cases in this group in both of these sensitivity analyses.

When we considered SC1 findings only, CRC incidence after SC1 was approximately 0.7 times lower among patients with low-risk findings at SC1 than in the general population and the detection rate of advanced PMPs at SC2 was 7%. Among those with high-risk findings at SC1, CRC incidence after SC1 was nearly three times higher than in the general population and the advanced PMP detection rate at SC2 was 16%. This shows that patients with low-risk findings at SC1 did not need ongoing surveillance, whereas those with high-risk findings at SC1 did. This is similar to the conclusions from the main analysis; the only difference is that this sensitivity analysis did not identify the small patient group for whom the need for ongoing surveillance was unclear.

Our study has limitations. The exclusion of patients who did not attend surveillance or could not be classified into a risk group might mean that our CRC risk estimates are higher than they would have been if we were able to include these patients. The observational and retrospective design meant that we lacked data on endoscopist performance indicators except for completeness and bowel preparation quality, and bowel preparation quality data were often missing due to the use of retrospective, routinely collected hospital data. We lacked complete data on reasons for follow-up visits, so we were unable to confirm whether they were for surveillance, symptom investigation or a positive screening test. Therefore, we cannot definitively say that our CRC risk estimates represent the risk of patients undergoing postpolypectomy surveillance, given the possibility of contamination from patients undergoing follow-up visits for other reasons. Another possibility is that patients could have had surveillance at hospitals not participating in the study.

The understanding, detection and classification of serrated polyps were limited during the study period. Our estimates of CRC incidence among patients compared with the general population might be underestimates because we excluded those with CRC before or at baseline, or at SC1, which did not apply to the general population. Additionally, we were unable to assess whether the study population was more healthy or less healthy than the general population, as data on comorbidities, anthropometric measures and lifestyle factors were unavailable. Therefore, health differences between the study population and the general population could have affected our SIR estimates. Results might have differed slightly if median intervals between baseline and SC1, and between SC1 and SC2, were consistent across the groups. Small group sizes with limited numbers of CRC cases prevented drawing conclusions about the need for second surveillance for the LR-HR group or understanding when the HR-HR group might have been able to stop surveillance.

Study strengths include its large size and nationwide design. We collected detailed data on patient, procedural and polyp characteristics at baseline and SC1. We examined the definitive outcome of CRC incidence. Excluding patients with baseline colonoscopies that were incomplete or of unknown completeness or had poor bowel preparation increased the applicability of the findings to modern practice. Surveillance regimens in our study population were mostly based on the 2002 UK surveillance guidelines,^17^ which had different risk group definitions and surveillance recommendations than the 2020 UK guidelines.^1^ Adherence to these guidelines was limited, so even large proportions of patients classified as having low-risk findings had surveillance. Therefore, we had the unique opportunity to examine CRC incidence after SC1 in patients with different combinations of low-risk and/or high-risk findings at baseline and SC1.

## Conclusion

Patients with high-risk findings at both baseline and SC1 needed a second surveillance visit, while those with low-risk findings at SC1 did not, regardless of their baseline findings. We could not draw conclusions for those with low-risk findings at baseline and high-risk findings at SC1, but this combination of findings appears to be uncommon. Our data suggest that there may be limited value in classifying patients using both baseline and SC1 findings, and that classification using SC1 findings alone may be sufficient to inform recommendations for the second surveillance visit, although this warrants further research.

## Supplementary material

10.1136/gutjnl-2024-334242 online supplemental file 1

10.1136/gutjnl-2024-334242 online supplemental file 2

## Data Availability

Data are available on reasonable request.
