# Supplementary material for: Colorectal cancer incidence after the first surveillance colonoscopy and the need for ongoing surveillance: a retrospective, cohort analysis
Source: Gut. 2025 Apr 5;74(9):e334242. doi: 10.1136/gutjnl-2024-334242 (PMC12418537; doi:10.1136/gutjnl-2024-334242)
Supplement: online supplemental file 1 [file gutjnl-74-9-s001.docx]

**Supplementary Table 1. Comparison of baseline characteristics between patients included in the analysis and those excluded because they did not attend surveillance or could not be classified into one of the four risk groups (n=21,895)**

| **Characteristic^a^** | **Included patients (n=10,508)**  n (%) | **Excluded patients (n=11,387)^b^**  n (%) |
| --- | --- | --- |
| Total |  |  |
| Classification of baseline findings |  |  |
| Low-risk | 6,835 (65.0) | 8,029 (70.5) |
| High-risk | 3,673 (35.0) | 2,413 (21.2) |
| Unclassifiable | 0 (0.0) | 945 (8.3) |
| Sex |  |  |
| Women | 4,310 (41.0) | 4,940 (43.4) |
| Men | 6,198 (59.0) | 6,447 (56.6) |
| Family history of cancer/CRC^c^ |  |  |
| No | 9,423 (89.7) | 10,819 (95.0) |
| Yes | 1,085 (10.3) | 568 (5.0) |
| Age at baseline visit, years |  |  |
| <55 | 2,411 (22.9) | 2,060 (18.1) |
| 55-64 | 3,436 (32.7) | 2,681 (23.5) |
| 65-74 | 3,468 (33.0) | 3,585 (31.5) |
| ≥75 | 1,193 (11.4) | 3,061 (26.9) |
| Year of baseline visit |  |  |
| 1984-1999 | 1,245 (11.8) | 946 (8.3) |
| 2000-2004 | 3,356 (31.9) | 3,594 (31.6) |
| 2005-2010 | 5,907 (56.2) | 6,847 (60.1) |
| Bowel preparation quality at baseline visit |  |  |
| Excellent or good | 3,874 (36.9) | 4,159 (36.5) |
| Satisfactory | 1,867 (17.8) | 2,506 (22.0) |
| Missing | 4,767 (45.4) | 4,722 (41.5) |
| No. of PMPs |  |  |
| 1 | 5,554 (52.9) | 6,832 (60.0) |
| 2 | 2,390 (22.7) | 2,567 (22.5) |
| 3 | 1,128 (10.7) | 1,048 (9.2) |
| 4 | 551 (5.2) | 474 (4.2) |
| ≥5 | 885 (8.4) | 466 (4.1) |
| No. of advanced PMPs |  |  |
| 0 | 4,783 (45.5) | 7,153 (62.8) |
| 1 | 4,575 (43.5) | 3,516 (30.9) |
| 2 | 861 (8.2) | 535 (4.7) |
| ≥3 | 289 (2.8) | 183 (1.6) |
| PMP size, mm^d^ |  |  |
| <10 | 4,922 (46.8) | 6,950 (61.0) |
| 10-19 | 3,325 (31.6) | 2,663 (23.4) |
| ≥20 | 2,226 (21.2) | 1,451 (12.7) |
| Unknown | 35 (0.3) | 323 (2.8) |
| Adenoma histology^e^ |  |  |
| Tubular | 5,825 (55.4) | 7,374 (64.8) |
| Tubulovillous | 3,568 (34.0) | 2,953 (25.9) |
| Villous | 612 (5.8) | 462 (4.1) |
| Unknown | 503 (4.8) | 598 (5.3) |
| Adenoma dysplasia^f^ |  |  |
| Low-grade | 8,896 (84.7) | 10,104 (88.7) |
| High-grade | 1,296 (12.3) | 838 (7.4) |
| Unknown | 316 (3.0) | 445 (3.9) |
| Proximal PMPs^g^ |  |  |
| No | 5,590 (53.2) | 6,245 (54.8) |
| Yes | 4,918 (46.8) | 5,142 (45.2) |

CRC: colorectal cancer. PMP: premalignant polyp.

^a^Comparing these characteristics between included and excluded patients, using the χ^2^ test, all comparisons had p-values ≤0.05.

^b^Excluded because they did not attend surveillance (n=10,104) or because information was missing on polyp characteristics needed to classify their baseline findings as low-risk or high-risk (n=945) or their first surveillance findings as low-risk or high-risk (n=338). In Figure 1, 1,799 patients were excluded because information was missing needed to classify their baseline findings as low-risk or high-risk; however, only 945 of these patients were included in the present comparison because the remaining 854 patients also had a suboptimal quality baseline colonoscopy. This comparison included only those who had a baseline colonoscopy of sufficient quality.

^c^Family history of cancer/CRC was defined as family history of cancer or CRC recorded by the patient’s endoscopist at an examination before or during the baseline visit.

^d^Defined according to the largest PMP recorded during the baseline visit.

^e^Defined according to the greatest degree of villousness recorded during the baseline visit; percentages were calculated using the number of patients with at least one adenoma as the denominator.

^f^Defined according to the highest grade of dysplasia recorded during the baseline visit; percentages were calculated using the number of patients with at least one adenoma as the denominator.

^g^Proximal was defined as proximal to the descending colon.

**Supplementary Table 2. Incidence of CRC and age-sex-standardised incidence ratios, by risk group, defining risk groups following the European post-polypectomy surveillance guidelines (n=10,328)**

| **Risk group^b^** | **No. of patients, n (%)** | **Person-years** | **Total no. of cases** | **Incidence rate per 100,000 person-years (95%CI)** | **At 3 years^a^** | | **At 5 years^a^** | | **Standardisation** | |
| --- | --- | --- | --- | --- | --- | --- | --- | --- | --- | --- |
|  |  |  |  |  | No. of cases | Cumulative incidence, % (95%CI)^c^ | No. of cases | Cumulative incidence, % (95%CI)^c^ | No. of expected cases | SIR (95%CI) |
| **After first surveillance, censored at any second surveillance visit^d^** | | | | | | | | | | |
| LR-LR | 4,173 (40.4) | 22,572 | 24 | 106 (71-159) | 10 | 0.3 (0.2-0.5) | 13^e^ | 0.4 (0.2-0.7) | 46 | 0.52 (0.34-0.78) |
| HR-LR | 5,197 (50.3) | 25,944 | 52 | 200 (153-263) | 17 | 0.4 (0.2-0.6) | 29^e^ | 0.9 (0.6-1.3) | 59 | 0.89 (0.66-1.16) |
| LR-HR | 250 (2.4) | 924 | 4 | 433 (162-1154) | 0 | “” | 2^e^ | 2.6 (0.6-10.3) | 2 | 1.96 (0.53-5.01) |
| HR-HR | 708 (6.9) | 2,381 | 13 | 546 (317-940) | 4 | 0.9 (0.3-2.5) | 10^e^ | 4.1 (2.1-7.8) | 6 | 2.14 (1.14-3.67) |
| **After second surveillance through the date of final censoring^f^** | | | | | | | | | | |
| LR-LR | 1,819 (34.4) | 11,698 | 13 | 111 (65-191) | 0 | “” | 3^g^ | 0.3 (0.1-0.9) | 25 | 0.53 (0.28-0.90) |
| HR-LR | 2,843 (53.7) | 19,211 | 36 | 187 (135-260) | 7 | 0.3 (0.1-0.6) | 15^g^ | 0.6 (0.4-1.1) | 47 | 0.77 (0.54-1.07) |
| LR-HR | 160 (3.0) | 921 | 0 | “” | 0 | “” | 0^g^ | “” | 2 | “” |
| HR-HR | 469 (8.9) | 3,238 | 10 | 309 (166-574) | 2 | 0.5 (0.1-1.9) | 5^g^ | 1.3 (0.6-3.2) | 9 | 1.15 (0.55-2.11) |

CI: confidence interval. CRC: colorectal cancer. HR-HR: high-risk, high-risk. HR-LR: high-risk, low-risk. LR-HR: low-risk, high-risk. LR-LR: low-risk, low-risk. SIR: standardised incidence ratio.

^a^For analyses of incidence after first surveillance, cumulative incidence data are shown for 3 and 5 years after first surveillance. For analyses of incidence after second surveillance, cumulative incidence data are shown for 3 and 5 years after second surveillance.

^b^Patients in the LR-LR group had low-risk findings at both baseline and first surveillance; those in the HR-LR group had high-risk findings at baseline and low-risk findings at first surveillance; those in the LR-HR group had low-risk findings at baseline and high-risk findings at first surveillance; and those in the HR-HR group had high-risk findings at both baseline and first surveillance. High-risk findings were defined as ≥1 ‘advanced’ premalignant polyp (adenoma ≥10mm or with high-grade dysplasia; serrated polyp ≥10mm or with any dysplasia) or ≥5 adenomas; findings not meeting these criteria were considered low-risk findings.

^c^Estimated using the Kaplan-Meier method.

^d^Each patient’s follow-up time was included from their first surveillance visit and censored at any second surveillance visit.

^e^The remaining CRC cases were diagnosed during the follow-up period starting 5 years after first surveillance (LR-LR, n=11; HR-LR, n=23; LR-HR, n=2; HR-HR, n=3).

^f^For those who attended ≥2 surveillance visits, each patient’s follow-up time was included from their second surveillance visit through the date of final censoring.

^g^The remaining CRC cases were diagnosed during the follow-up period starting 5 years after second surveillance (LR-LR, n=10; HR-LR, n=21; LR-HR, n=0; HR-HR, n=5).

**Supplementary Table 3. Incidence of CRC and age-sex-standardised incidence ratios, by risk group, excluding incompletely excised baseline lesions (n=10,506)^a^**

| **Risk group^c^** | **No. of patients, n (%)** | **Person-years** | **Total no. of cases** | **Incidence rate per 100,000 person-years (95%CI)** | **At 3 years^b^** | | **At 5 years^b^** | | **Standardisation** | |
| --- | --- | --- | --- | --- | --- | --- | --- | --- | --- | --- |
|  |  |  |  |  | No. of cases | Cumulative incidence, % (95%CI)^d^ | No. of cases | Cumulative incidence, % (95%CI)^d^ | No. of expected cases | SIR (95%CI) |
| **After first surveillance, censored at any second surveillance visit^e^** | | | | | | | | | | |
| LR-LR | 6,588 (62.7) | 35,342 | 35 | 99 (71-138) | 14 | 0.2 (0.1-0.4) | 17^f^ | 0.3 (0.2-0.5) | 72 | 0.48 (0.34-0.67) |
| HR-LR | 3,306 (31.5) | 15,308 | 41 | 268 (197-364) | 12 | 0.4 (0.3-0.8) | 26^f^ | 1.4 (0.9-2.1) | 37 | 1.11 (0.80-1.50) |
| LR-HR | 247 (2.4) | 902 | 5 | 555 (231-1,332) | 0 | “” | 3^f^ | 4.0 (1.3-12.0) | 2 | 2.51 (0.81-5.86) |
| HR-HR | 365 (3.5) | 1,140 | 6 | 526 (236-1,172) | 2 | 0.9 (0.2-3.7) | 3^f^ | 2.0 (0.6-7.1) | 3 | 2.07 (0.76-4.50) |
| **After second surveillance through the date of final censoring^g^** | | | | | | | | | | |
| LR-LR | 3,079 (57.1) | 20,677 | 25 | 121 (82-179) | 3 | 0.1 (0.0-0.3) | 9^h^ | 0.4 (0.2-0.8) | 45 | 0.56 (0.36-0.82) |
| HR-LR | 1,890 (35.1) | 12,511 | 22 | 176 (116-267) | 4 | 0.2 (0.1-0.6) | 9^h^ | 0.6 (0.3-1.1) | 32 | 0.68 (0.43-1.03) |
| LR-HR | 163 (3.0) | 952 | 1 | 105 (15-746) | 0 | “” | 0^h^ | “” | 2 | 0.43 (0.01-2.39) |
| HR-HR | 256 (4.8) | 1,787 | 10 | 560 (301-1,040) | 1 | 0.5 (0.1-3.2) | 4^h^ | 2.0 (0.7-5.1) | 5 | 2.07 (0.99-3.80) |

CI: confidence interval. CRC: colorectal cancer. HR-HR: high-risk, high-risk. HR-LR: high-risk, low-risk. LR-HR: low-risk, high-risk. LR-LR: low-risk, low-risk. PMP: premalignant polyp. SIR: standardised incidence ratio.

^a^CRCs deemed likely to have arisen from incompletely excised baseline premalignant polyps (PMPs) were excluded (6 of 151 total CRCs). PMPs seen at first surveillance that were deemed likely to have been incompletely excised at baseline were excluded from the risk group classification (101 of 7,158 total PMPs seen at first surveillance); this led to the additional exclusion of two patients, compared to the main analysis, because their findings at first surveillance could no longer be classified as low-risk or high-risk.

^b^For analyses of incidence after first surveillance, cumulative incidence data are shown for 3 and 5 years after first surveillance. For analyses of incidence after second surveillance, cumulative incidence data are shown for 3 and 5 years after second surveillance.

^c^Patients in the LR-LR group had low-risk findings at both baseline and first surveillance; those in the HR-LR group had high-risk findings at baseline and low-risk findings at first surveillance; those in the LR-HR group had low-risk findings at baseline and high-risk findings at first surveillance; and those in the HR-HR group had high-risk findings at both baseline and first surveillance. High-risk findings were defined as ≥2 PMPs of which ≥1 was ‘advanced’ (adenoma ≥10mm or with high-grade dysplasia; serrated polyp ≥10mm or with any dysplasia), ≥5 PMPs, or ≥1 large (≥20mm) non-pedunculated PMP; findings not meeting these criteria were considered low-risk findings.

^d^Estimated using the Kaplan-Meier method.

^e^Each patient’s follow-up time was included from their first surveillance visit and censored at any second surveillance visit.

^f^The remaining CRC cases were diagnosed during the follow-up period starting 5 years after first surveillance (LR-LR, n=18; HR-LR, n=15; LR-HR, n=2; HR-HR, n=3).

^g^For those who attended ≥2 surveillance visits, each patient’s follow-up time was included from their second surveillance visit through the date of final censoring.

^h^The remaining CRC cases were diagnosed during the follow-up period starting 5 years after second surveillance (LR-LR, n=16; HR-LR, n=13; LR-HR, n=1; HR-HR, n=6).

**Supplementary Table 4. Incidence of CRC and age-sex-standardised incidence ratios, by risk group, excluding patients whose baseline visit occurred before 2000 (n=9,263)**

| **Risk group^b^** | **No. of patients, n (%)** | **Person-years** | **Total no. of cases** | **Incidence rate per 100,000 person-years (95%CI)** | **At 3 years^a^** | | **At 5 years^a^** | | **Standardisation** | |
| --- | --- | --- | --- | --- | --- | --- | --- | --- | --- | --- |
|  |  |  |  |  | No. of cases | Cumulative incidence, % (95%CI)^c^ | No. of cases | Cumulative incidence, % (95%CI)^c^ | No. of expected cases | SIR (95%CI) |
| **After first surveillance, censored at any second surveillance visit^d^** | | | | | | | | | | |
| LR-LR | 5,780 (62.4) | 30,318 | 30 | 99 (69-142) | 12 | 0.2 (0.1-0.4) | 15^e^ | 0.3 (0.2-0.6) | 62 | 0.49 (0.33-0.69) |
| HR-LR | 2,906 (31.4) | 13,216 | 34 | 257 (184-360) | 10 | 0.4 (0.2-0.8) | 23^e^ | 1.4 (0.9-2.1) | 32 | 1.07 (0.74-1.49) |
| LR-HR | 226 (2.4) | 831 | 4 | 481 (181-1,282) | 0 | “” | 2^e^ | 2.6 (0.7-10.3) | 2 | 2.23 (0.61-5.70) |
| HR-HR | 351 (3.8) | 1,071 | 6 | 560 (252-1,247) | 3 | 1.4 (0.4-4.3) | 5^e^ | 4.1 (1.5-11.0) | 3 | 2.19 (0.80-4.76) |
| **After second surveillance through the date of final censoring^f^** | | | | | | | | | | |
| LR-LR | 2,568 (55.9) | 14,725 | 13 | 88 (51-152) | 2 | 0.1 (0.0-0.3) | 8^g^ | 0.5 (0.2-0.9) | 31 | 0.41 (0.22-0.71) |
| HR-LR | 1,639 (35.7) | 9,713 | 16 | 165 (101-269) | 4 | 0.3 (0.1-0.7) | 9^g^ | 0.7 (0.4-1.3) | 25 | 0.65 (0.37-1.06) |
| LR-HR | 149 (3.2) | 796 | 1 | 126 (18-892) | 0 | “” | 0^g^ | “” | 2 | 0.54 (0.01-3.02) |
| HR-HR | 241 (5.2) | 1,547 | 9 | 582 (303-1,118) | 1 | 0.5 (0.1-3.4) | 4^g^ | 2.1 (0.8-5.5) | 4 | 2.17 (0.99-4.11) |

CI: confidence interval. CRC: colorectal cancer. HR-HR: high-risk, high-risk. HR-LR: high-risk, low-risk. LR-HR: low-risk, high-risk. LR-LR: low-risk, low-risk. SIR: standardised incidence ratio.

^a^For analyses of incidence after first surveillance, cumulative incidence data are shown for 3 and 5 years after first surveillance. For analyses of incidence after second surveillance, cumulative incidence data are shown for 3 and 5 years after second surveillance.

^b^Patients in the LR-LR group had low-risk findings at both baseline and first surveillance; those in the HR-LR group had high-risk findings at baseline and low-risk findings at first surveillance; those in the LR-HR group had low-risk findings at baseline and high-risk findings at first surveillance; and those in the HR-HR group had high-risk findings at both baseline and first surveillance. High-risk findings were defined as ≥1 ‘advanced’ premalignant polyp (adenoma ≥10mm or with high-grade dysplasia; serrated polyp ≥10mm or with any dysplasia) or ≥5 adenomas; findings not meeting these criteria were considered low-risk findings.

^c^Estimated using the Kaplan-Meier method.

^d^Each patient’s follow-up time was included from their first surveillance visit and censored at any second surveillance visit.

^e^The remaining CRC cases were diagnosed during the follow-up period starting 5 years after first surveillance (LR-LR, n=15; HR-LR, n=11; LR-HR, n=2; HR-HR, n=1).

^f^For those who attended ≥2 surveillance visits, each patient’s follow-up time was included from their second surveillance visit through the date of final censoring.

^g^The remaining CRC cases were diagnosed during the follow-up period starting 5 years after second surveillance (LR-LR, n=5; HR-LR, n=7; LR-HR, n=1; HR-HR, n=5).

**Supplementary Table 5. Detection rates of advanced PMPs at second surveillance, by risk group, in sensitivity analyses**

| **Sensitivity analysis** | **Risk group** | **Total no. of patients** | **No. of patients attending a second surveillance visit, n (%)** | **Interval from first to second surveillance in years, median (IQR)** | **Detection rate of advanced PMPs at second surveillance, % (95%CI)^a^** |
| --- | --- | --- | --- | --- | --- |
| Excluding incompletely excised baseline lesions^b^ | LR-LR^c,d^ | 6,588 | 3,079 (46.7) | 3.1 (2.4-4.6) | 5.3 (4.6-6.2) |
|  | HR-LR^c,d^ | 3,306 | 1,890 (57.2) | 3.0 (2.1-3.4) | 8.6 (7.4-10.0) |
|  | LR-HR^c,d^ | 247 | 163 (66.0) | 2.3 (1.3-3.2) | 12.3 (7.7-18.3) |
|  | HR-HR^c,d^ | 365 | 256 (70.1) | 1.9 (1.1-3.1) | 14.5 (10.4-19.4) |
| Excluding patients whose baseline visit occurred before 2000 | LR-LR^c,d^ | 5,780 | 2,568 (44.4) | 3.1 (2.5-4.5) | 5.3 (4.5-6.3) |
|  | HR-LR^c,d^ | 2,906 | 1,639 (56.4) | 3.0 (2.3-3.4) | 9.1 (7.7-10.6) |
|  | LR-HR^c,d^ | 226 | 149 (65.9) | 2.6 (1.2-3.3) | 10.7 (6.3-16.9) |
|  | HR-HR^c,d^ | 351 | 241 (68.7) | 1.8 (1.1-3.1) | 16.2 (11.8-21.5) |
| Defining risk groups based on first surveillance findings only | ‘SC1 LR’^d,e^ | 9,859 | 4,944 (50.1) | 3.1 (2.3-4.1) | 6.8 (6.1-7.5) |
|  | ‘SC1 HR’^d,e^ | 649 | 446 (68.7) | 2.0 (1.1-3.1) | 15.5 (12.2-19.2) |

HR-HR: high-risk, high-risk. HR-LR: high-risk, low-risk. IQR: interquartile range. LR-HR: low-risk, high-risk. LR-LR: low-risk, low-risk. PMP: premalignant polyp. ‘SC1 HR’: high-risk at first surveillance. ’SC1 LR’: low-risk at first surveillance.

^a^Advanced PMPs were defined as an adenoma ≥10mm or with high-grade dysplasia, or a serrated polyp ≥10mm or with any dysplasia.

^b^Colorectal cancers (CRCs) deemed likely to have arisen from incompletely excised baseline premalignant polyps (PMPs) were excluded (6 of 151 total CRCs). PMPs seen at first surveillance that were deemed likely to have been incompletely excised at baseline were excluded from the risk group classification (101 of 7,158 total PMPs seen at first surveillance); this led to the additional exclusion of two patients, compared to the main analysis, because their findings at first surveillance could no longer be classified as low-risk or high-risk. Additionally, PMPs seen at second surveillance that were deemed likely to have been incompletely excised at baseline were excluded from calculations of advanced PMP detection rates (30 of 4,064 total PMPs seen at second surveillance).

^c^Patients in the LR-LR group had low-risk findings at both baseline and first surveillance; those in the HR-LR group had high-risk findings at baseline and low-risk findings at first surveillance; those in the LR-HR group had low-risk findings at baseline and high-risk findings at first surveillance; and those in the HR-HR group had high-risk findings at both baseline and first surveillance.

^d^High-risk findings were defined as ≥2 PMPs of which ≥1 was ‘advanced’ (adenoma ≥10mm or with high-grade dysplasia; serrated polyp ≥10mm or with any dysplasia), ≥5 PMPs, or ≥1 large (≥20mm) non-pedunculated PMP; findings not meeting these criteria were considered low-risk findings.

^e^Patients in the ‘SC1 LR’ group had low-risk findings at first surveillance with any baseline findings; those in the ‘SC1 HR’ group had high-risk findings at first surveillance with any baseline findings.

**Supplementary Table 6. Incidence of CRC and age-sex-standardised incidence ratios, by risk group, defining risk groups based on first surveillance findings only (n=10,508)**

| **Risk group^b^** | **No. of patients, n (%)** | **Person-years** | **Total no. of cases** | **Incidence rate per 100,000 person-years (95%CI)** | **At 3 years^a^** | | **At 5 years^a^** | | **Standardisation** | |
| --- | --- | --- | --- | --- | --- | --- | --- | --- | --- | --- |
|  |  |  |  |  | No. of cases | Cumulative incidence, % (95%CI)^c^ | No. of cases | Cumulative incidence, % (95%CI)^c^ | No. of expected cases | SIR (95%CI) |
| **After first surveillance, censored at any second surveillance visit^d^** | | | | | | | | | | |
| ‘SC1 LR’ | 9,859 (93.8) | 50,551 | 78 | 154 (124-193) | 27 | 0.3 (0.2-0.5) | 45^e^ | 0.7 (0.5-0.9) | 109 | 0.72 (0.57-0.89) |
| ‘SC1 HR’ | 649 (6.2) | 2,142 | 14 | 654 (387-1,104) | 4 | 1.0 (0.4-2.7) | 9^e^ | 4.2 (2.1-8.4) | 5 | 2.71 (1.48-4.55) |
| **After second surveillance through the date of final censoring^f^** | | | | | | | | | | |
| ‘SC1 LR’ | 4,944 (91.7) | 33,002 | 48 | 145 (110-193) | 8 | 0.2 (0.1-0.4) | 19^g^ | 0.5 (0.3-0.8) | 77 | 0.63 (0.46-0.83) |
| ‘SC1 HR’ | 446 (8.3) | 2,941 | 11 | 374 (207-675) | 1 | 0.3 (0.0-1.9) | 4^g^ | 1.2 (0.5-3.2) | 8 | 1.43 (0.71-2.55) |

CI: confidence interval. CRC: colorectal cancer. SIR: standardised incidence ratio. ‘SC1 HR’: high-risk at first surveillance. ‘SC1 LR’: low-risk at first surveillance.

^a^For analyses of incidence after first surveillance, cumulative incidence data are shown for 3 and 5 years after first surveillance. For analyses of incidence after second surveillance, cumulative incidence data are shown for 3 and 5 years after second surveillance.

^b^Patients in the ‘SC1 LR’ group had low-risk findings at first surveillance with any baseline findings; those in the ‘SC1 HR’ group had high-risk findings at first surveillance with any baseline findings. High-risk findings were defined as ≥2 premalignant polyps (PMPs) of which ≥1 was ‘advanced’ (adenoma ≥10mm or with high-grade dysplasia; serrated polyp ≥10mm or with any dysplasia), ≥5 PMPs, or ≥1 large (≥20mm) non-pedunculated PMP; findings not meeting these criteria were considered low-risk findings.

^c^Estimated using the Kaplan-Meier method.

^d^Each patient’s follow-up time was included from their first surveillance visit and censored at any second surveillance visit.

^e^The remaining CRC cases were diagnosed during the follow-up period starting 5 years after first surveillance (‘SC1 LR’, n=33; ‘SC1 HR’, n=5).

^f^For those who attended ≥2 surveillance visits, each patient’s follow-up time was included from their second surveillance visit through the date of final censoring.

^g^The remaining CRC cases were diagnosed during the follow-up period starting 5 years after second surveillance (‘SC1 LR’, n=29; ‘SC1 HR’, n=7).
