# Supplementary figures and images for: Colorectal cancer incidence after the first surveillance colonoscopy and the need for ongoing surveillance: a retrospective, cohort analysis
Source: Gut. 2025 Apr 5;74(9):e334242. doi: 10.1136/gutjnl-2024-334242 (PMC12418537; doi:10.1136/gutjnl-2024-334242)

## Slide 1
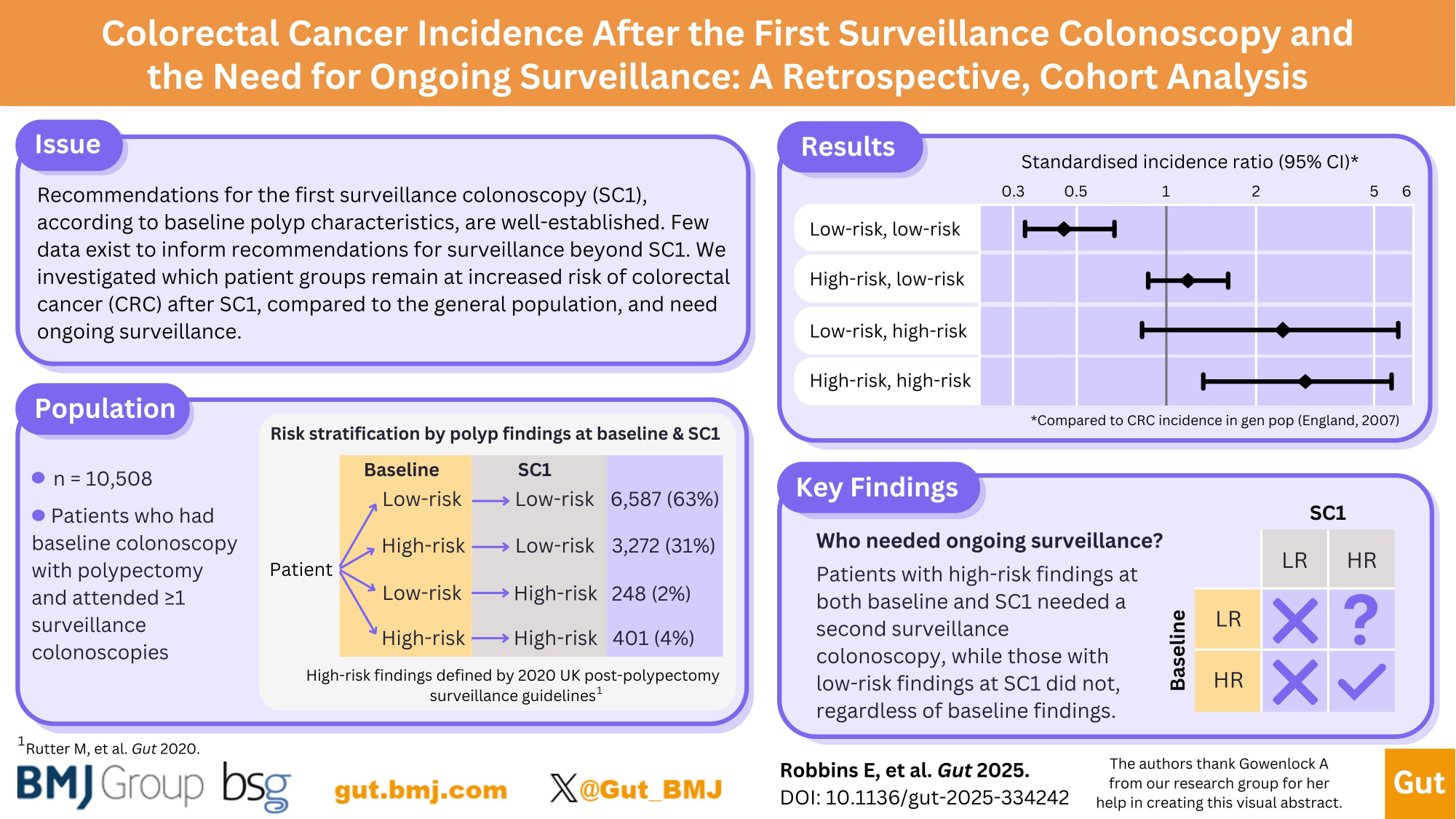

Supplement: online supplemental file 2 [file gutjnl-74-9-s002.pptx]
